# Limited effects of short-term dietary L-glutamate supplementation on intestinal morphology of 100 g hybrid striped bass (*Morone saxatilis* ♀× *Morone chrysops* ♂)

**DOI:** 10.3389/fimmu.2026.1799411

**Published:** 2026-05-05

**Authors:** Karina L. Hissen, Michael F. Criscitiello

**Affiliations:** 1 Comparative Immunogenetics Laboratory, Department of Veterinary Pathobiology, Texas A&M University, College Station, TX, United States; 2 Department of Microbial Pathogenesis and Immunology, College of Medicine, Texas A&M Health Science Center, Bryan, TX, United States

**Keywords:** aquaculture, glutamate, hybrid striped bass, intestinal mucosa, nutrition

## Abstract

**Introduction:**

The aquaculture industry has been exploring new methods to enhance fish production, including using amino acids as supplements to boost growth and health. L-glutamate (Glu) is one of the most abundant amino acids in both animal and plant protein sources, but it has been considered nutritionally non-essential in fish diets because it can be produced endogenously. We recently reported that dietary supplementation of Glu within a purified amino acid diet increased growth in non-immune challenged hybrid striped bass that were 25 grams and modulated intestinal mucosal immunity that were immune stimulated. Although this shows that Glu can be an effective immunonutrient, no studies have assessed its effects on larger hybrid striped bass. This study aims to evaluate the impact of dietary supplementation of Glu on 100-gram hybrid striped bass that were not immune stimulated, testing the hypothesis that increasing amounts of Glu added to a purified diet would improve growth and intestinal morphology.

**Methods:**

Purified diets containing 0%, 1%, 3%, 5%, and 8% Glu were fed to 100 g hybrid striped bass for four weeks and compared with a group fed 60% fishmeal serving as a baseline control group. After the dietary intervention, serum and mucosa samples were analyzed to assess amino acid concentration, while middle intestine samples were evaluated histologically.

**Results:**

Four weeks of Glu supplementation did not have an effect on growth performance in 100 g fish, where all groups increased in weight by approximately 16% (P > 0.05). Intestinal mucosa Glu concentration increased with dietary level, reaching about 15.7 nmol per mg of tissue in fish fed 8% Glu (P < 0.001). Modest effects were seen in the decrease of goblet cells per villus in fish fed 0% Glu (P < 0.05), but there were no differences in intestinal morphology among the groups (P > 0.05).

**Discussion:**

While purified diets may not be an effective approach to improve growth or intestinal morphology in these hybrid striped bass, Glu may still be necessary for goblet cell abundance. Further research is warranted to inform amino acid supplementation strategies for specific developmental stages to achieve cost-effective growth and disease resistance.

## 1 Introduction

As the global human population increases, aquatic animals offer a nutritious source of high-quality protein, with world aquaculture production reaching 144.3 million tons and generating a total of $313 billion in sales in 2022 (1). This was the first time aquaculture had surpassed capture fisheries, demonstrating the impact of the Food and Agriculture Organization of the United Nations’ (FAO) Blue Transformation global strategy to expand aquatic food systems sustainably (1). Toward this goal, aquaculture producers’ nutrition strategies have focused on alternatives to fishmeal (FM), a primary protein source made from small pelagic fish (2). While FM is the most nutritious protein source in fish diets, it presents several drawbacks, such as limited fish to make FM. FM also presents economic risks: feed costs can range from 40-70% of an aquaculture production because FM is the most expensive protein component of the diet (3), with reported values of fishmeal at $1,743.02/ton (4). To mitigate cost risk and reduce the need to capture fish to make FM, diets are often formulated to utilize plant-protein sources, such as soybean meal (SBM) (5). The aquaculture industry has been utilizing SBM since the 1980s; it is cost-effective at $354.38/ton (6). While SBM is a promising alternative protein source to FM, its inclusion has not been fully optimized for carnivorous fish, as it has a different amino acid (AA) composition from FM (3, 7, 8). Typically, carnivorous fish have a higher requirement for protein, and with higher stocking densities, the need to replace FM is essential to meet sustainable goals (3).

These low FM diets have been shown to have adverse effects on growth performance and intestinal health in some carnivorous fish (9). For example, Atlantic salmon (*Salmo salar*) fed SBM to partially replace FM developed subacute enteritis in the distal intestine after three weeks, which progressed for the remainder of the six-week feeding experiment (10). Chinook (*Oncorhynchus tshawytscha*) and Coho Salmon (*Oncorhynchus kisutch*) fed soybean and cottonseed products to substitute FM saw reduced weight gain and increased mortality after 14 weeks and 20 weeks, respectively (11). In Atlantic cod (*Gadus morhua*), the total replacement of FM using SBM, soy protein concentrate, and wheat gluten reduced growth (12) and displayed necrotic damage in all gut segments (13). In European sea bass (*Dicentrarchus labrax*), complete vegetable meal replacement after 90 days reduced growth and modulated the gut microbiome (14). Moreover, FM alternatives have been shown to reduce feed intake, digestibility, and feed efficiency, each of which impacts growth (15, 16). This could be due to the composition of SBM which affects the palatability of diets, leading to different feeding behaviors, as seen in Chinook salmon (*Oncorhynchus tshawytscha*) (11, 17), Abernathy salmon (*Oncorhynchus nerka*) (18), Asian sea bass (*Lates calcarifer*) (19, 20), obscure puffer (*Takifugu fasciatus*) (21), and red drum (*Sciaenops ocellatus*) (22, 23). However, little is known about the impact of FM replacement on the carnivorous fish, hybrid striped bass (HSB; *Morone chrysops* x *Morone saxatilis*), which is a cross between piscivorous striped bass and white bass (24).

The original HSB cross is called the “palmetto bass”, where the female is the striped bass and the male is the white bass, while the reciprocal cross, called the “sunshine bass,” is a cross between a male striped bass and a female white bass (5). The hybridization was first accomplished by Robert Stevens in 1965 to mediate the rapid decline of the wild striped bass population (25). Hybridization improved growth, disease resistance, feed conversion rates, survival rates, temperature tolerance, and tolerance to water with low dissolved oxygen levels (26–31). Annual production of HSB has increased since 1990, where it jumped from 636,000 kg to 3.8 million kg in 1997 (32), and peaked in 2000 at 5.1 million kg (33). By 2018, HSB production had become a major aquaculture industry in the U.S., ranking #3 in sales ($100 million) after catfish (*Siluriformes*) and trout (*Oncorhynchus*) (34). In many states, including Texas (35), HSB farming is currently the second largest aquacultural enterprise behind only catfish farming. There are three phases of HSB production, and the process takes between 18–24 months: Phase 1, where larvae will develop a simple digestive tract by 4–5 days old, and are held in in fry ponds until 30–45 days (5, 27, 36); Phase 2, where 1 gram (g) HSB are fed frequently until producer-desired weight based on stocking densities of 125 to 225 g (36); and Phase 3, the grow out stage where HSB remain in ponds until reaching market size of 680 to 1130 g (27, 33). Some producers have eliminated Phase 2 to save time, labor and eliminate handling stress from grading and transferring (36).

Most protein nutrition research in aquaculture is conducted with freshwater omnivorous species that are fed diets containing significant plant-based feed ingredients. These species do not display appreciably compromised growth performance and are therefore less dependent on FM and other animal-sourced feedstuffs (37, 38). As mentioned, SBM has been used as the major alternative to FM in aquaculture because of its excellent nutritional values, high levels of crude protein, well-balanced profiles for most amino acids (AAs), and high nutrient digestibility (39). Non-essential amino acid (NEAAs), like L-glutamate (Glu), have been overlooked for it is produced endogenously in tissues (40–42). While Glu is abundant in proteins of animal and plant origins, such as FM, poultry by-product meal, and SBM (7, 8), its AA composition differs between the two protein sources. It was reported in by Li et al. (7) that peptide-bound Glu was more in Menhaden FM compared to SBM at approximately 6.01% and 4.17% respectively (7). In the same study, immediately absorbable Glu, free AA, was approximately 1.31 mg/g and 0.18 mg/g in FM and SBM respectively (7). Another study compared AA composition of various sources of FM, including menhaden, Peruvian anchovy, and Southeast Asian miscellaneous marine fishes to SBM (8). They reported that total Glu (peptide-bound and free AA) was more in FM, regardless of source (51.28 to 59.73 g/kg), compared to SBM (42.15 g/kg) (8). There are limited studies on Glu deficiency in carnivorous fish, but one study in juvenile little yellow croaker (*Larimichthys polyactis*) revealed that a 0% Glu diet shortens villi length and increases villi damage (43). Another observation was an incidental finding from a facility accident mid-trial, in which juvenile HSB were exposed to abrupt changes in water quality overnight (44). There were no survivors from the 0% Glu fed fish, in contrast to the 52-54% survival rate seen in the 1-5% Glu fed fish (44). These results could indicate that dietary glutamate supplementation is required for intestinal health and survival.

Glu is more actively oxidized in the intestine of HSB, generating ATP in the tissue to utilize for cellular function (45). Glu’s ability to bind to specialized taste receptors for umami perception (46) could make diets be more palatable for carnivores like HSB and increase intestinal health through its utilization. This highlights the importance of understanding Glu’s role in the nutrition of teleost fish like HSB. We have previously reported that 8% Glu supplementation in a purified AA diet enhanced growth in juvenile HSB (approximately 29.9 g to 38.9 g) after one week and possibly increased goblet cell distribution (47). This present study continues to investigate the effects of Glu supplementation in 100 g HSB. The study expanded the diet profiles to include five Glu concentrations at 0% (negative control), 1%, 3%, 5%, and 8%. We also included a group fed 60% fish meal (FM), a conventional control diet for its high-quality protein and performance baseline (48).

The primary objective of this study is to investigate the impact of dietary Glu on intestinal morphology. The hypothesis tested is that Glu supports growth and enhances intestinal mucosal immunity parameters in HSB by increasing the available mucosal surface area, thereby enabling a greater distribution of goblet cells. The findings of this research will not only provide essential basic knowledge about AA metabolism in carnivorous fish but also help develop better, environmentally friendly aquafeeds for fish and other aquatic species.

## 2 Materials and methods

This study (AUP # IACUC 2023-0247) was reviewed and approved by the Institutional Animal Care and Use Committee at Texas A&M University (College Station, TX, USA). All animal procedures were conducted in accordance with the Animal Welfare Act and Regulations of the United States Department of Agriculture.

### 2.1 HSB husbandry and sample collection

#### 2.1.1 Animals and housing

HSBs used in this study were obtained in April 2025 from Keo Fish Farm (Keo, Arkansas, USA) and were all from the same spring-spawned cohort (hatched the previous spring in 2024). Upon arrival, HSB were housed in a recirculating aquaculture system (39, 47, 49–51) that was maintained with increased water flow to enable greater water exchange and support higher biomass. Our experimental setup consisted of 36 tanks, each with two fish, containing 38 gallons of water, maintained as described in Hissen et al. (47), with the exception that dissolved oxygen was measured daily (47). The facility had a 14-hour photo period, during which the light cycle spanned from 6:00 AM to 8:00 PM each day. The fish were acclimated in our system for two weeks by consuming approximately 3% of their body weight (BW) per day. During the first week of acclimation, HSB were fed a diet with 60% FM twice daily (**Table 1**). In the second week of acclimation, the diet shifted to a purified crystalline AA diet containing 3% Glu (**Table 1**), which aligns with the nutritional profile of 60% FM, and was provided three times daily. Purified diets offer precise supplementation, with crystalline AA >98% pure and in their free form, to be readily absorbed, in contrast to FM, which requires time for active digestion to break down proteins into free AAs.

**Table 1:** The formulation of 60% FM and the purified AA diets as fed used during the 4-week supplementation period.

Diet formulation						
Ingredients^a^ (g/kg)	60% FM	0% Glu	1% Glu	3% Glu	5% Glu	8% Glu
Fish oil^b^	43	110	110	110	110	110
Soy oil^c^	10	10	10	10	10	10
Dextrinized starch^d^	200	200	200	200	200	200
Cellulose^e^	105.66	151.77	147.82	139.93	132.04	120.21
Carboxymethyl cellulose (CMC)^f^	30	30	30	30	30	30
Vitamin premix	0.998^g^	1.055^h^	1.055^h^	1.055^h^	1.055^h^	1.055^h^
Mineral premix	3.09^i^	77.04^j^	77.04^j^	77.04^j^	77.04^j^	77.04^j^
Non-AA nitrogenous substances	7.28^k^	11.20^l^	11.20^l^	11.20^l^	11.20^l^	11.20^l^
Menhaden fish meal^m^	600	0.0	0.0	0.0	0.0	0.0
Amino acid (AA) mix^n^ (see below)	0	348.94	352.89	360.78	368.67	380.50
Water	60	60	60	60	60	60
Total	1060	1000	1000	1000	1000	1000
AA mix^n^	g/kg	g/kg	g/kg	g/kg	g/kg	g/kg
Arg	0	20.2	20.2	20.2	20.2	20.2
Asn	0	12.5	12.5	12.5	12.5	12.5
Asp	0	18.3	18.3	18.3	18.3	18.3
Cys	0	3.7	3.7	3.7	3.7	3.7
Gln	0	20.1	20.1	20.1	20.1	20.1
Gly	0	23.5	23.5	23.5	23.5	23.5
His	0	7.8	7.8	7.8	7.8	7.8
Ile	0	13.3	13.3	13.3	13.3	13.3
Leu	0	24.8	24.8	24.8	24.8	24.8
Lys-HCl	0	30.7	30.7	30.7	30.7	30.7
Met	0	10.9	10.9	10.9	10.9	10.9
Phe	0	12.8	12.8	12.8	12.8	12.8
Pro	0	20.6	20.6	20.6	20.6	20.6
Ser	0	14.0	14.0	14.0	14.0	14.0
Thr	0	13.8	13.8	13.8	13.8	13.8
Trp	0	3.9	3.9	3.9	3.9	3.9
Tyr	0	10.4	10.4	10.4	10.4	10.4
Val	0	16.7	16.7	16.7	16.7	16.7
Tau	0	2.5	2.5	2.5	2.5	2.5
Ala	0	68.4	62.4	50.3	38.2	20.0
Glu	0	0.0	10.0	30.0	50.0	80.0

^a^ Values are expressed on an as-fed basis.

^b^ Menhaden fish oil (Paragon, Illinois, USA).

^c^ Nutrioli pure soybean oil (Ragasa, N.L., Mexico).

^d^ Maltodextrin (Amazon.com, Seattle, MA, USA).

^e^ Microcrystaline cellulose 102 (Blue Diamond Growers, California, USA).

^f^ Sodium carboxy methyl cellulose (Pro Supply Outlet, California, USA).

^g^ Providing the following vitamins for FM diet (mg/kg): retinyl (vitamin A), 23.06; cholecalciferol (vitamin D_3_), 20.24; DL-α-tocopheryl (vitamin E), 188.7; menadione (vitamin K_3_), 12; vitamin C, 300.0; DL-calcium pantothenate (vitamin B_5_), 103.7; myo-inositol, 150.0; niacin, 106.9; pyridoxine (vitamin B_6_), 26.8; vitamin B_2_ 27.1; thiamine (vitamin B_1_), 32.2; biotin, 1.4; folic acid, 5.9; vitamin B_12_, 0.1.

^h^ Providing the following vitamins for each purified diet (mg/kg diet): retinyl (vitamin A), 23.06; cholecalciferol (vitamin D_3_), 20.24; DL-α-tocopheryl (vitamin E), 200.0; menadione (vitamin K_3_), 12.0; vitamin C, 300.0; DL-calcium pantothenate (vitamin B_5_), 109.0; myo-inositol, 150.0; niacin, 140.0; pyridoxine (vitamin B_6_), 30.4; vitamin B_2_, 30.0; thiamine (vitamin B_1_), 32.6; biotin, 1.5; folic acid, 6.0; vitamin B_12_, 0.2.

^i^ Providing the following minerals for FM diet (mg/kg): NaCl, 3050; CuSO_4_·5H_2_O, 26; CoCl·6H_2_O, 3.3; KI, 6.3.

^j^ Providing the following minerals premix for each purified diet (mg/kg diet): CaHPO_4_·2H_2_O, 33700; NaCl, 15300; MgSO_4_·7H_2_O, 14000; KCl, 13140; Chromium(III) chloride, 7.3; CuSO_4_·5H_2_O, 35; FeSO_4_·7H_2_O, 498; MnSO_4_·4H_2_O, 82; Na_2_SeO_3_, 3; ZnSO_4_·7H_2_O, 258; sodium molybdate, 0.26; sodium fluoride 1.3; CoCl·6H_2_O, 5.2; KI, 7.8; nickel chloride, 2.2.

^k^ Providing the following N-containing chemicals for FM diet (g/kg diet): betaine 4.25; inosine 5 monophosphate, 3; creatine, 0.72, carnitine, 0.08.

^l^ Providing the following N-containing chemicals for each purified diet (g/kg diet): choline chloride, 2.4; betaine, 5; Inosine 5 monophosphate, 3; creatine, 0.72, carnitine, 0.08.

^m^ Omega fishmeal, Menhaden (Omega Protein, Houston, Texas).

^n^ Crystalline AAs (Ajinomoto, Tokyo, Japan).

#### 2.1.2 Diets

Both 60% FM and the purified AA diets were prepared at Texas A&M University’s Aquacultural and Research Teaching Facility (52). The formulation of the diets is listed in **Table 1**, where the purified AA diets have all AAs used in their L-form, except for glycine. To ensure that the experimental diets were isonitrogenous, L-alanine was added to the purified diets in place of glutamic acid (Glu). All diets were formulated to provide recommended proportions of essential amino acids, vitamins, and minerals for HSB, consistent with National Research Council (NRC) standards for HSB (53). For the purified diets, the following dry ingredients were mixed in a Hobart L-800 mixer (Hobart Inc., Troy, OH) for at least 45 minutes (min) to create a “common mix” that will be allocated to each purified diet: dextrinized starch, carboxymethyl cellulose, vitamin and mineral premixes, non-AA nitrogenous substances, and the AA mix excluding alanine and Glu. For each purified diet, the “common mix”, cellulose, alanine, and Glu were mixed using a V-type powder mixer (P-K Blend Master, Patterson-Kelley LLC, PA, USA) for 30 min. The dry ingredients were loaded into a Hobart A-200 mixer (Hobart Inc., Troy, OH), followed by the addition of fish and soybean oil. The diet was then mixed for 15 minutes to achieve a homogeneous blend. Water was added at the end of this mixing period to achieve a semi-moist mixture, which was then blended for an additional 15 minutes in the Hobart A-200 mixer. Following this, diets were extruded through a 3-mm diameter die plate at the end of a meat chopper attachment connected to the Hobart mixer. The resultant diet strands were allowed to dry for at least 24 hours at room temperature before being further dried in an oven at 37 °C until all diets reached a similar dry matter content, approximately 95%, ensuring consistent nutrient delivery. Strands were cut into approximately ¼-inch pieces using a blender (Ninja Brand, Needham, MA). The prepared pellets were stored at –20 °C in plastic zip-lock bags until use. The 60% FM diet was prepared by first finely grinding menhaden FM using a VEVOR 1000g electric grain mill grinder (VEVOR, Hangzhou, China). It was then combined with the other dry ingredients using a V-type powder mixer, following the same process described above.

#### 2.1.3 Experimental trial

Fish were briefly held in a tared container, and two fish of approximately 97.5 grams (g) were randomly assigned to one of 6 tanks per diet group, which includes five Glu concentrations at 0% (negative control; 0 g/kg), 1% (10 g/kg), 3% (30 g/kg), 5% (50 g/kg), and 8% (80 g/kg), and a positive control group with 60% FM. Feed pellets were manually administered to each tank to ensure accurate delivery of designated rations. In the event of mortality, feeding rates were recalculated according to the number of surviving fish so that feed was provided on a per-fish basis. During the 4 weeks, HSB were fed thrice daily a percentage of their body weight (BW; composite weight in grams of all fish in a tank). HSB were fed 3.5% of their BW on each of days 0–13, then 4% of their BW on days 14–28. These feeding rates were chosen based on the results of a preliminary study (data not shown). This was adjusted daily, anticipating HSB growth of 1 g/day as shown in He et al. (51).

#### 2.1.4 Sample collection

Necropsies and tissue sampling were conducted one hour after the HSB were fed. Collection one hour postprandially was selected based on a kinetic study using radiolabeled Glu, in which approximately 500 nmol of Glu was transported in the intestine from the mucosa to the serosa of 250 g HSB in one hour (data not shown). From each conscious fish, approximately 1 mL of whole blood was collected from the caudal vein using a 1-mL non-heparinized syringe. The sample was immediately centrifuged at 10,000 × g for 1 minute, serum collected, then placed in liquid nitrogen and stored at –80 °C until biochemical analysis. Immediately after blood sampling, HSBs were euthanized with freshly prepared 400 ppm Syncaine^®^ (tricaine methanesulfonate; MS-222; Syndel, WA, USA) buffered to pH 7.5 with sodium bicarbonate, and unresponsiveness was confirmed as described in Hissen et al. (47). Subsequently, fish were weighed directly on a tared scale before a secondary confirmatory method, pithing, was performed to ensure death. The intestine was collected, and the lumen content was removed before a portion of the middle intestine was collected for histological analysis. The remaining segments were then butterflied open to be washed with oxygenated in (95% O_2_/5% CO_2_) Ca^+^-free Krebs–Henseleit bicarbonate buffer (KHB; pH 7.4; 119 mM NaCl, 4.8 mM KCl, 1.2 mM MgSO_4_, 1.2 mM KH_2_PO_4_, and 25 mM NaHCO_3_, pH 7.4) containing 20 mM Hepes (pH 7.4), 5 mM glucose and 0.5% fatty acid-free bovine serum albumin. The mucosa was then collected as described in Hissen et al. (47), then placed in liquid nitrogen and stored at –80 °C until AA analysis.

### 2.2 Analysis of AAs

One fish per tank (n = 6 fish per diet) was chosen randomly for analysis of free AAs in the serum and intestinal mucosa were quantified using a Waters Alliance HPLC system (Milford, MA, USA), employing precolumn derivatization with o-phthaldialdehyde (OPA) as previously described by Dai et al. (54). The serum samples were processed and analyzed as described in Hissen et al. (47) for plasma. For the mucosa, the tissue was homogenized with a pestle in 1.7 mL Eppendorf tubes to ensure the 25 mg subset collected for analysis was an accurate representative of the tissue. This subset of tissue was acidified with 250 µL of 1.5 mol/L HClO_4_, vortexed, and further homogenized with a pestle. The acidified samples were transferred into a 5 mL Eppendorf tube, mixed with 1.25 mL of HPLC-grade water, neutralized with 125 µL of 2 mol/L K_2_CO_3_, and vortexed again. Following neutralization, the samples were centrifuged at 10,000 × g for 1 min using an Eppendorf 5920R centrifuge, and the supernatant was collected for AA analysis. This supernatant was analyzed in the same way as for the serum samples. AA values below the detection limit were treated as zero for graphing but excluded from statistical analyses.

### 2.3 Histology

**Figure 1:**
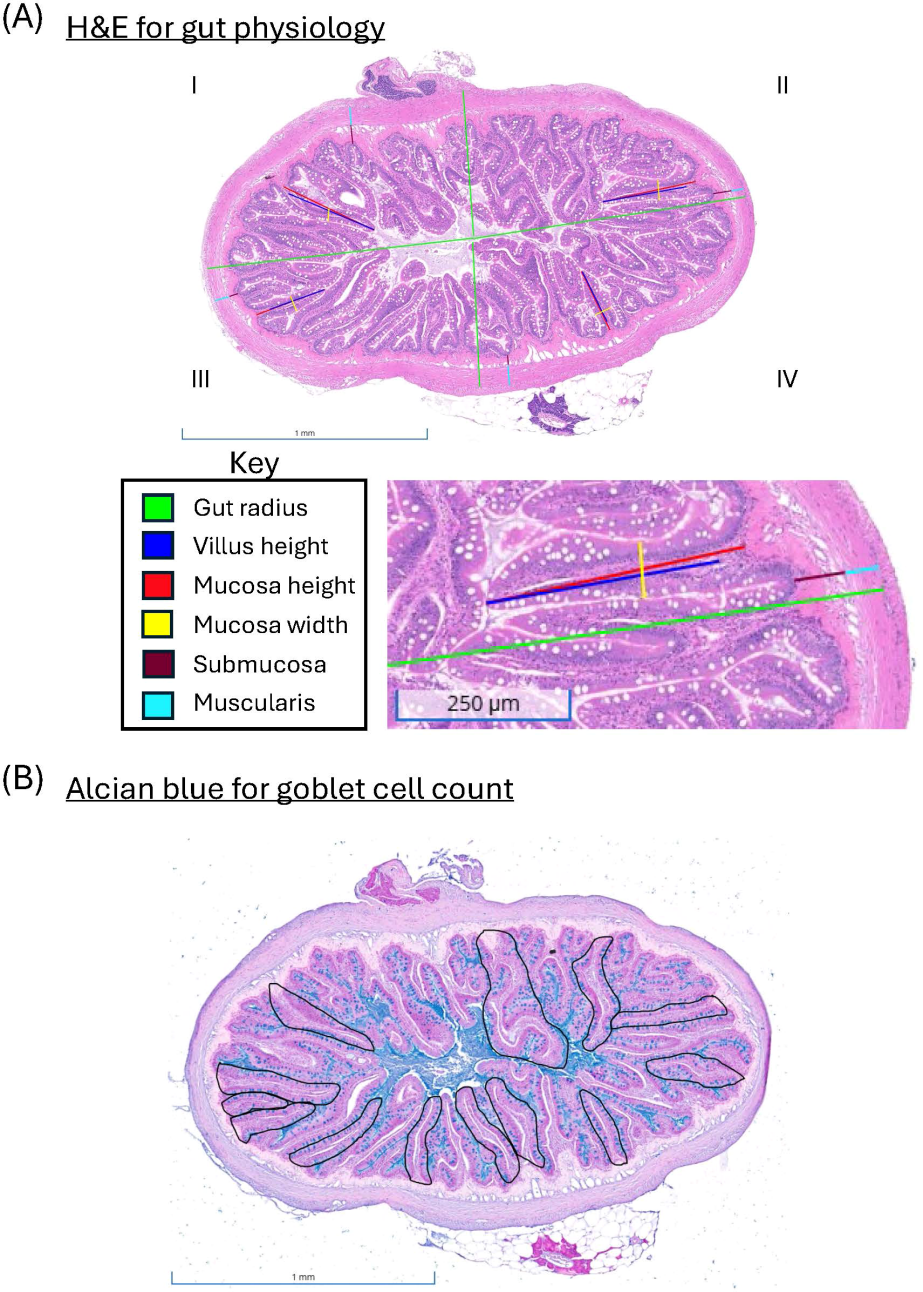
Parameters for morphology measurements **(A)** and goblet cell counts **(B)** of the middle intestine in fish fed 60% FM. Panel A) displays how four quadrants are created by measuring the radius on the two shortest sides and the two longest sides (green). Within each quadrant, the height of the villus (dark blue) and mucosa (red), the width of the mucosa (yellow), the thickness of the submucosa (maroon), and the muscularis (teal) were measured. These four representing replicates were averaged to determine a representative measurement for the organ of that fish. Panel B) shows the 13 villi measured to determine the number of goblet cells per villus.

To assess intestinal mucosa morphology, middle intestine sections were chosen from the same fish used in the AA analysis experiments described above (n = 6 fish per diet). The intestine pieces were placed in Carnoy’s Solution (60% ethanol, 30% chloroform, and 10% glacial acetic acid) for 45 minutes (55, 56), then rinsed with 70% ethanol to stop the fixation process and stored in 70% ethanol until processing. These samples were sent to the Veterinary Medicine and Biomedical Sciences (VMBS) Core Histology Laboratory at Texas A&M, where they were embedded in paraffin, cut into 4-µm cross sections with a rotary microtome, and placed on charged slides. For each gut sample, adjacent serial sections were stained with hematoxylin and eosin (H&E) and Alcian Blue, pH 2.5. Every 500 µm, consecutive sections were skipped to broaden the sample for analysis and provide three cross-sections. Slides were scanned and analyzed using Concentriq by Proscia. One of the three sections per sample was chosen for the following measurements: the radius of the organ, the height of the villus and mucosa, the width of the mucosa, the submucosal thickness, and the muscularis thickness. Due to the sections not all being circular, we refined the parameters described in Li et al. (57), as follows (**Figure 1A**):

1. Radius: Measurements from the center of the organ to the muscularis before the serosa were taken on the two shortest sides and the two longest sides.
2. Villus height: Within each of the four quadrants formed from the radius measurement, villi were randomly sampled, but only those that were well oriented, straight, or gently curved with no kinks were included in the random sampling. Villus height was measured from apical tip to the villus-crypt junction.
3. Mucosa height: On the selected villi, mucosa height was measured from the apical tip to the basal interface with the submucosa.
4. Width of mucosa: On the selected villi, width measurements were taken mid-height from one lateral epithelial side to the other, including the lamina propria.
5. Submucosal thickness: Within each of the four quadrants formed from measuring the radius, measurements were taken from the base of the mucosa (basal to the crypt) to the apical edge of the muscularis externa.
6. Muscularis thickness: Within each of the four quadrants formed from measuring the radius, measurements were taken from the submucosa-muscularis layer to the basal edge of the serosa.

For the above measurements, the four replicates were averaged to determine a representative value for the organ of that fish. Additionally, goblet cell counts were manually counted from 13 villi, adapted from Blick et al. (58). While we did not cut open the intestine to preserve its integrity, we excluded the opening of the crypts as a criterion. To maximize the usable tissue area across biological replicates, we defined qualifying villi based on distinguishable crypt structures, with the epithelial surfaces being primarily intact (**Figure 1B**).

### 2.4 Calculations and statistical analysis

All data are presented as means ± SEM. For growth performance, weight gain rate (WGR) was calculated as the percent of difference between the final and initial BW (g) divided by the initial BW (WGR = 100*(final BW – initial BW)/initial BW). The feed conversion ratio (FCR) was calculated as the total feed intake divided by the weight gain in each tank (FCR = total feed intake (g)/total weight gain (g)). The gain-to-feed ratio (G:F) was calculated as the average daily gain divided by the feed intake in each tank (G: F = average daily gain (g)/feed intake (g)). Statistical analyses were performed using JMP Pro 17 software (SAS Institute, Cary, NC), with a significance level of α = 0.05. Data on growth performance parameters were analyzed by both linear and quadratic regression analysis. Additionally, one-way ANOVA was performed to compare the effects of purified diets on growth performance, AA content in tissues, and histological parameters. If ANOVA results were significant, SNK multiple comparison was applied to compare the differences in means. Additionally, to compare the effects of the purified diets to our positive control diet, 60% FM, we performed Dunnett’s test, which directly tests treatment control differences.

## 3 Results

### 3.1 Growth performance

The growth performance of 100 g HSB after a 28-day supplementation period in this present study is shown in **Table 2**. There were no significant differences observed in growth parameters between the various Glu purified diets (P > 0.05). Additionally, no significant differences were detected when Dunnett’s test was performed to compare the growth parameters of the purified diets with those of the control diet, 60% FM (P > 0.05).

**Table 2:** Growth performance of 100 g HSB after a 28-day supplementation period.

Variable	60% FM	0% Glu	1% Glu	3% Glu	5% Glu	8% Glu	^1^ *P-Values*	^2^ *Linear*	^3^ *Quadratic*
Initial BW (g/fish)	97.7 ± 1.48	97.8 ± 0.44	95.1 ± 1.33	99.7 ± 2.73	98 ± 0.71	96.5 ± 1.30	*0.301*	*0.979*	*0.262*
Final BW (g/fish)	115 ± 2.78	111 ± 1.91	111 ± 3.25	118 ± 7.75	111 ± 2.05	114 ± 3.64	*0.690*	*0.618*	*0.585*
WGR (%)	17.8 ± 2.50	13.3 ± 1.67	16.6 ± 2.40	18.4 ± 6.04	13.6 ± 1.49	18.3 ± 2.63	*0.687*	*0.519*	*0.968*
FCR	8.33 ± 1.67	10.4 ± 1.19	8.56 ± 1.16	12.2 ± 4.06	11.9 ± 2.40	7.94 ± 1.19	*0.700*	*0.731*	*0.199*
G:F	0.14 ± 0.02	0.10 ± 0.01	0.13 ± 0.02	0.15 ± 0.05	0.10 ± 0.02	0.14 ± 0.02	*0.623*	*0.635*	*0.965*

Values are means ± SEM, n = 6 tanks per diet group (2 fish per tank).

^1^ Probability for one-way ANOVA for each growth parameter among the purified diets in italics.

^2^ Probability for linear regression analysis for each growth parameter among the purified diets in italics.

^3^ Probability for quadratic analysis for each growth parameter among the purified diets in italics.

### 3.2 AA concentrations

#### 3.2.1 Serum

The concentrations of only a few AAs in the serum of 100 g HSB were affected by Glu supplementation (**Table 3**). In order of most significance to least significance, glycine, taurine, arginine, alanine, and tyrosine were modulated by Glu supplementation among the purified diets (P < 0.05). Glycine concentrations in the serum were highest in HSB supplemented with 3% Glu, and lowest in HSB supplemented with 0% Glu (P = 0.001). Contrary to expectations, HSB fed 3% Glu had 1.54 times more glycine in the serum compared to HSB fed 60% FM (P = 0.002). For taurine, concentrations in serum were highest in HSB supplemented with 0%, 1%, and 3% Glu, while they were the lowest in HSB fed 8% Glu (P = 0.004). Additionally, HSB fed 0%, 1%, and 3% Glu diets had almost double the amount of taurine in serum compared to HSB fed 60% FM (P < 0.05). Next, arginine serum concentrations were highest in HSB supplemented with 5% and 8% Glu, and lowest in HSB fed with 3% Glu (P = 0.011). For alanine, HSB supplemented with 3% Glu had the highest serum levels, while HSB supplemented with 8% Glu had the lowest (P = 0.012). Furthermore, HSB fed the purified diets, excluding those supplemented with 8% Glu, had substantially higher alanine levels compared to HSB fed 60% FM (P < 0.05). These levels almost tripled in HSB fed 0%, 1% and 5% Glu, and even quadrupled in HSB fed 3% Glu (P < 0.001). Lastly, tyrosine serum concentrations were highest in HSB fed 0% Glu, while the lowest were seen in HSB supplemented with 3% Glu (P = 0.041). Moreover, HSB supplemented with 0% Glu also had higher amounts of tyrosine compared to HSB fed 60% FM (P <0.05).

**Table 3:** Concentrations (nmol/ml) of amino acids (AAs) in the serum of 100 g HSB after a 28-day supplementation period.

AA	60% FM	0% Glu	1% Glu	3% Glu	5% Glu	8% Glu	*P-Values* ^1^
Gly	516 ± 62.1	489 ± 32.7^c^	704 ± 19.2^ab^	797 ± 51.5^a*^	688 ± 65.0^ab^	556 ± 54.8^bc^	*0.001*
Tau	673 ± 111	1237 ± 102^a*^	1228 ± 114^a*^	1246 ± 155^a*^	973 ± 47.3^ab^	736 ± 41.8^b^	*0.004*
Arg	95.8 ± 13.0	118 ± 13.4^ab^	105 ± 11.1^ab^	67.9 ± 4.82^b^	144 ± 18.5^a^	136 ± 20.7^a^	*0.011*
Ala	541 ± 63.9	1789 ± 224^ab*^	1762 ± 219^ab*^	2288 ± 381^a*^	1390 ± 207^ab*^	990 ± 98.2^b^	*0.012*
Tyr	50.9 ± 4.59	98.1 ± 12.5^a*^	75.6 ± 14.5^ab^	47.7 ± 9.69^b^	67.4 ± 9.33^ab^	64.5 ± 5.2^ab^	*0.041*
Glu	62.5 ± 19.4	78.5 ± 13.6	155 ± 31.8*	138 ± 33.2	76.9 ± 12.1	90.6 ± 12.7	*0.068*
Gln	371 ± 41.9	702 ± 77.7*	690 ± 57.4*	775 ± 87.8*	598 ± 67.1	510 ± 67.1	*0.123*
Cit	23.4 ± 4.05	34.8 ± 5.95	29.7 ± 5.26	16.4 ± 2.79	27.9 ± 5.26	29.3 ± 4.57	*0.134*
Phe	73.9 ± 6.40	113 ± 10.2	112 ± 10.0	80.8 ± 12.2	124 ± 16.9*	106 ± 7.08	*0.143*
Orn	6.52 ± 2.14	8.59 ± 3.06	7.13 ± 1.03	ND^†^	14.2 ± 2.77	6.93 ± 2.85	*0.186*
Trp	24.6 ± 1.77	31.8 ± 4.09	29.1 ± 5.39	20.4 ± 4.87	31.1 ± 2.28	32.1 ± 2.54	*0.244*
Met	58.3 ± 4.17	99.2 ± 20.3	104 ± 20.9	63.0 ± 23.9	125 ± 12.9	114 ± 21.8	*0.281*
Thr	103 ± 7.25	282 ± 32.5*	222 ± 29.1	225 ± 40.5	269 ± 49.6*	173 ± 34.8	*0.299*
Asp	8.20 ± 4.24	15.8 ± 4.57	26.9 ± 6.97	34.7 ± 12.2*	20.1 ± 5.94	16.7 ± 5.04	*0.366*
Ile	119 ± 12.5	214 ± 20.2	172 ± 25.7	154 ± 26.5	214 ± 34.0	166 ± 28.4	*0.388*
Leu	178 ± 15.6	362 ± 30.0*	294 ± 34.4	281 ± 43.8	362 ± 44.6*	278 ± 50.5	*0.388*
Ser	103 ± 13.5	250 ± 37.8*	236 ± 17.8*	220 ± 27.4	264 ± 40.7*	181 ± 40.4	*0.484*
Asn	130 ± 21.0	290 ± 26.7	313 ± 36.3	375 ± 57.2*	351 ± 79.7*	246 ± 56.3	*0.499*
β-Ala	2.56 ± 0.24	3.01 ± 0.53	3.61 ± 0.86	5.92 ± 0.94	5.77 ± 2.01	6.79 ± 3.06	*0.501*
Lys	112 ± 18.7	165 ± 32.1	159 ± 23.2	201 ± 28.4	233 ± 45.6*	175 ± 35.0	*0.524*
Val	245 ± 31.5	503 ± 51.9*	404 ± 58.1	393 ± 75.7	465 ± 85.0	380 ± 81.8	*0.707*
His	366 ± 90.8	110 ± 16.6*	122 ± 16.0*	96.9 ± 14.9*	100 ± 28.1*	86.2 ± 20.3*	*0.748*

Values are means ± SEM, n = 6 fish per diet.

^1^ Probability for one-way ANOVA for each AA among the purified diets in italics.

^a-c^ Means in a row without a common superscript letter differ (*P* < 0.05) as analyzed by one-way ANOVA and the SNK test. FM not included in ANOVA analysis.

^*^ Dunnet’s test used to compare effects of purified diets to 60% FM (*P* < 0.05).

^†^ Orn concentration within 3% Glu was not detectable (ND) and was not included for statistical analysis.

Other noteworthy observations include that serum ornithine levels were undetectable in all samples from HSB fed 3% Glu. Additionally, Dunnett’s test revealed a handful of AAs that were significantly higher in serum concentration in HSB fed purified diets compared to the 60% FM control diet (P < 0.05). However, the one-way ANOVA did not reveal any difference among the purified diets. For example, HSB fed 0% Glu had higher serum concentrations of glutamine (+89%), leucine (+103%), valine (+105%), serine (+143%), and threonine (+174%). HSB fed 1% Glu had higher serum concentrations of glutamine (+86%), serine (+129%), and Glu (+148%). HSB fed 3% Glu had higher serum concentrations of glutamine (+109%), asparagine (+189%), and aspartate (+323%). HSB fed 5% Glu had higher serum concentrations of phenylalanine (+68%), leucine (+103%), lysine (+108%), threonine (+151%), serine (+156%), and asparagine (+170%). Unexpectedly, all HSB fed the purified diets had lower histidine levels compared to HSB fed 60% FM.

#### 3.2.2 Intestinal mucosa

In the intestinal mucosa (**Table 4**), glycine, glutamate, ornithine, arginine, and serine were modulated from Glu supplementation among the purified diets. Glycine mucosa concentrations were highest in HSB fed 5% and 8% Glu compared to the other treatment groups (P = 0.0002). The most meaningful observation was that Glu concentrations increased in the mucosa with increasing dietary Glu levels, showing a 4-fold increase between the HSB fed 0% Glu and 8% Glu (P = 0.0003), which supports our hypothesis. Additionally, HSB fed 0% Glu had significantly lower Glu in the intestinal mucosa compared to HSB fed 60% FM. The AA that demonstrated the next statistical significance was ornithine, where the highest intestinal mucosa concentration was seen in HSB fed 5% Glu and the lowest was seen in HSB fed 0% and 3% Glu (P = 0.020). No significant differences were observed relative to HSB fed 1% and 8% Glu. For arginine, HSB fed 5% Glu and 8% Glu had significantly more arginine in their mucosa compared to HSB fed 3% Glu, which had the lowest concentration (P = 0.027). Mean concentrations HSB fed 5% Glu and 8% were not statistically different compared to HSB fed 0% and 1% Glu. All HSB fed the purified diets had less arginine in their mucosa compared to the HSB fed 60% FM (P < 0.05). Lastly, while the one-way ANOVA revealed significant treatment effects on serine levels within the mucosa (P = 0.028), the SNK test indicated that no specific groups fed the purified diets differed strongly enough from one another. However, the Dunnett’s test revealed that HSB fed 0%, 1%, and 3% Glu had lower levels of serine compared to HSB fed the control diet of 60% FM. Similar to serum AA concentrations, Dunnett’s test revealed a handful of AAs that were significantly lower in concentration in HSB fed purified diets compared to the control diet, 60% FM (P < 0.05). However, the one-way ANOVA did not reveal any difference among the purified diets.

**Table 4:** Concentrations (nmol per mg of tissue) of amino acids (AAs) in the intestinal mucosa of 100 g HSB after a 28-day supplementation period.

AA	60% FM	0% Glu	1% Glu	3% Glu	5% Glu	8% Glu	^1^ *P-Values*
Gly	8.62 ± 0.80	5.89 ± 1.01^b^	7.11 ± 0.64^b^	6.00 ± 0.68^b^	9.56 ± 0.98^a^	10.9 ± 0.45^a^	*0.0002*
Glu	10.1 ± 0.62	3.92 ± 0.18^c*^	5.99 ± 1.38^bc^	6.68 ± 1.42^bc^	11.1 ± 1.78^ab^	15.7 ± 2.61^a^	*0.0003*
Orn	0.11 ± 0.02	0.06 ± 0.01^b^	0.07 ± 0.01^ab^	†0.03 ± 0.01^b^	0.13 ± 0.03^a^	0.08 ± 0.02^ab^	*0.020*
Arg	7.59 ± 0.59	3.90 ± 0.43^ab*^	3.53 ± 0.59^ab*^	2.09 ± 0.57^b*^	4.70 ± 0.56^a*^	4.32 ± 0.61^a*^	*0.027*
Ser	9.73 ± 0.75	4.64 ± 0.41^a*^	5.50 ± 0.94^a*^	4.61 ± 0.76^a*^	7.59 ± 0.70^a^	7.66 ± 1.20^a^	*0.028*
Met	2.88 ± 0.22	1.94 ± 0.15*	1.85 ± 0.27*	1.50 ± 0.28*	2.28 ± 0.18	2.52 ± 0.35	*0.083*
Lys	9.06 ± 0.51	4.91 ± 0.47*	4.62 ± 0.72*	3.23 ± 0.84*	5.85 ± 0.88*	4.90 ± 0.81*	*0.215*
Phe	4.35 ± 0.31	2.66 ± 0.23*	2.38 ± 0.35*	2.01 ± 0.45*	3.00 ± 0.34	3.05 ± 0.46	*0.289*
Leu	8.67 ± 0.63	5.78 ± 0.48	5.43 ± 0.73*	4.45 ± 0.99*	6.70 ± 0.63	6.67 ± 1.12	*0.295*
Val	8.06 ± 0.69	4.28 ± 0.60*	4.80 ± 0.96	4.01 ± 0.98*	6.14 ± 0.87	6.53 ± 1.43	*0.315*
Tau	23.0 ± 2.26	17.1 ± 3.20	21.0 ± 2.69	16.9 ± 2.12	22.5 ± 2.52	22.6 ± 2.15	*0.320*
Asp	5.92 ± 0.40	3.97 ± 0.92	5.54 ± 1.55	5.15 ± 1.74	6.11 ± 1.53	8.98 ± 2.44	*0.339*
Gln	5.87 ± 0.41	3.73 ± 0.44	4.08 ± 0.55	3.81 ± 0.83	5.06 ± 0.50	5.04 ± 0.65	*0.350*
Ile	5.54 ± 0.44	3.03 ± 0.37*	2.92 ± 0.50*	2.56 ± 0.64*	3.86 ± 0.50	4.01 ± 0.83	*0.356*
Trp	1.22 ± 0.12	0.75 ± 0.06*	0.58 ± 0.08*	0.50 ± 0.13*	0.74 ± 0.09*	0.70 ± 0.12*	*0.369*
His	1.62 ± 0.27	0.74 ± 0.12*	0.83 ± 0.14*	0.91 ± 0.25	1.12 ± 0.14	1.09 ± 0.21	*0.507*
Tyr	4.06 ± 0.27	2.52 ± 0.26*	2.33 ± 0.35*	2.21 ± 0.47*	2.93 ± 0.39	2.98 ± 0.57	*0.617*
Thr	9.12 ± 0.50	5.64 ± 1.04	6.30 ± 1.50	6.03 ± 1.74	8.07 ± 1.58	8.37 ± 1.72	*0.626*
Cit	0.07 ± 0.02	0.08 ± 0.02	0.11 ± 0.02	0.17 ± 0.03	0.13 ± 0.04	0.15 ± 0.08	*0.643*
Asn	4.38 ± 0.37	2.52 ± 0.52	2.79 ± 0.60	3.10 ± 0.96	3.87 ± 0.59	3.25 ± 0.49	*0.662*
β-Ala	0.05 ± 0.00	0.05 ± 0.02	0.05 ± 0.01	0.07 ± 0.02	0.07 ± 0.02	0.06 ± 0.02	*0.810*
Ala	12.5 ± 0.97	12.0 ± 1.80	13.4 ± 1.56	11.7 ± 2.66	12.6 ± 0.88	11.1 ± 1.37	*0.906*

Values are means ± SEM, n = 6 fish per diet.

^1^ Probability for one-way ANOVA for each AA among the purified diets in italics.

^a-c^ Means in a row without a common superscript letter differ (*P* < 0.05) as analyzed by one-way ANOVA and the SNK test. FM not included in ANOVA analysis.

^*^ Dunnet’s test used to compare effects of purified diets to 60% FM (*P* < 0.05).

^†^ Orn concentration from one sample within 3% Glu was not detectable (ND) and was not included for statistical analysis (n = 5).

### 3.3 Histology

**Figure 2:**
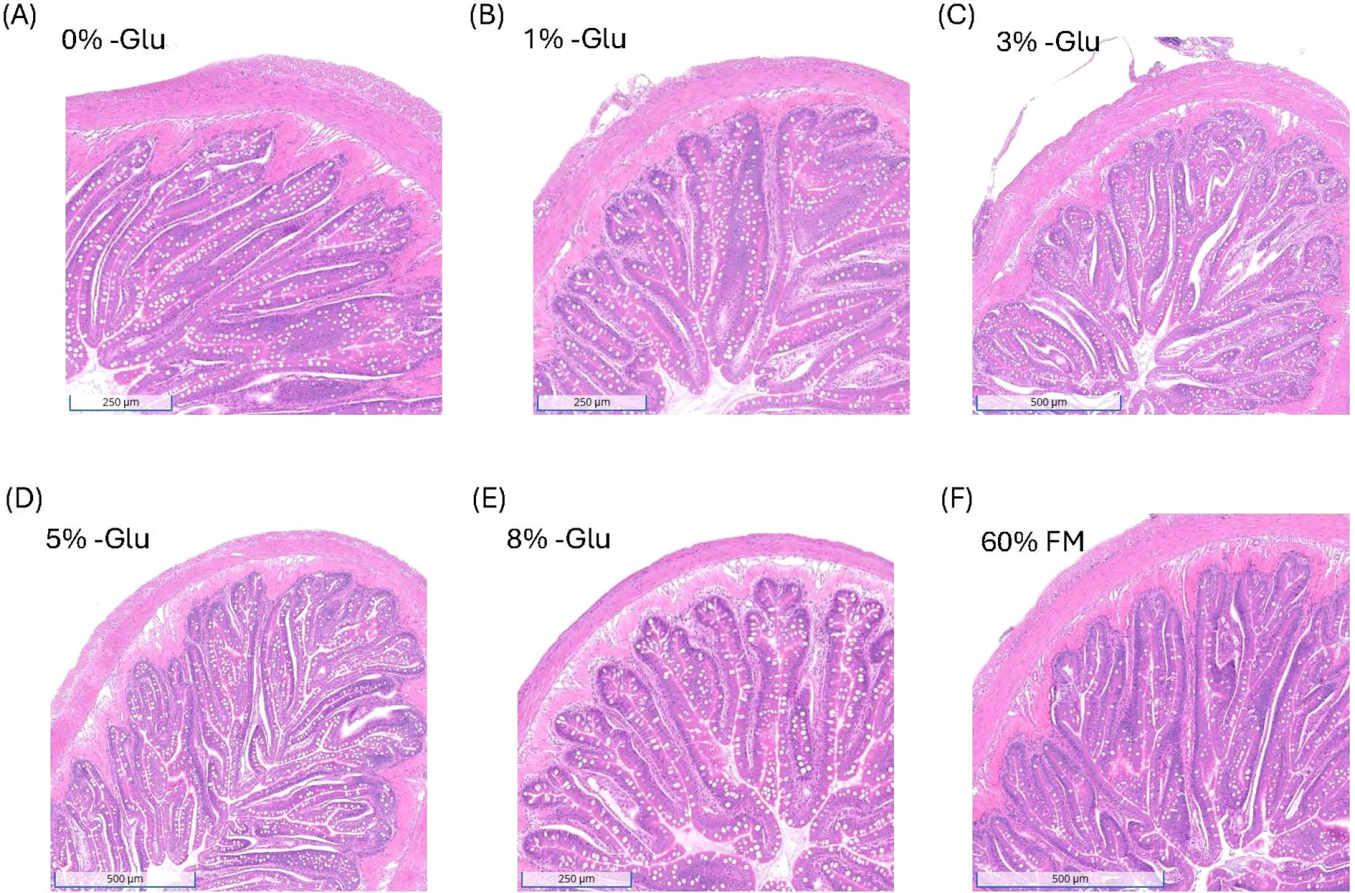
Morphology did not differ in 100 g HSB after 28 days of supplementation with various concentrations of glutamate compared to 60%-FM. Screenshots of a H&E-stained intestinal section were taken for each diet, **(A)** 0% Glu, **(B)** 1% Glu, **(C)** 3% Glu, **(D)** 5% Glu, **(E)** 8% Glu, and **(F)** 60% FM, using the Concentriq software. Note different scale bars, different magnifications shown to show the largest histological representation.

**Figure 3:**
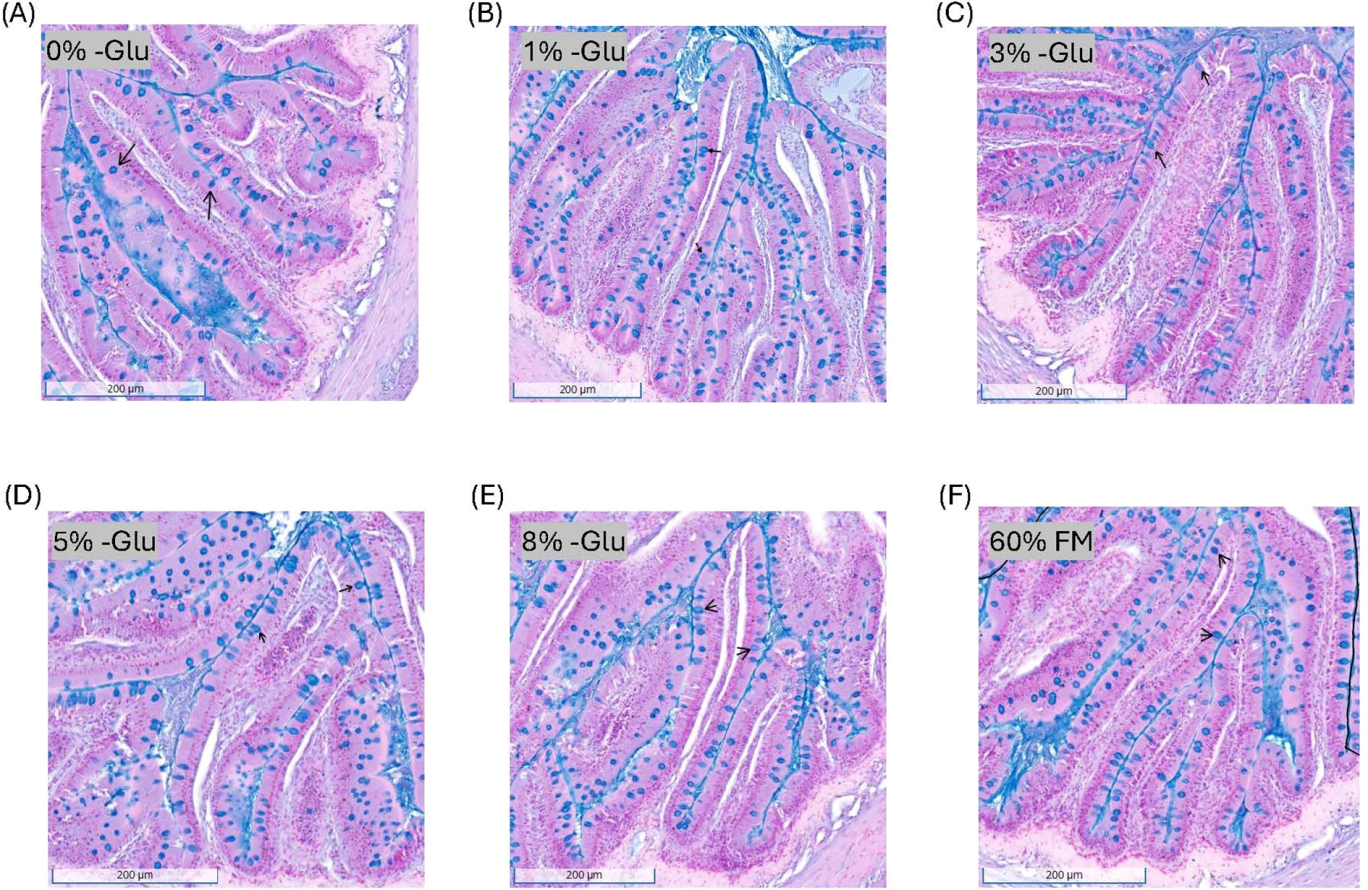
Goblet cell distribution along the villi of the middle intestine in 100 g HSB did not increase with glutamate supplementation. Screenshots of an Alcian blue stained intestinal section were taken for each diet, **(A)** 0% Glu, **(B)** 1% Glu, **(C)** 3% Glu, **(D)** 5% Glu, **(E)** 8% Glu, and **(F)** 60% FM, using Concentriq software. Black arrows indicate representative goblet cells that were counted in the mucosal epithelium.

Data on the histological evaluation of gut morphology in the middle intestine are shown in **Table 5**. The radius of the gut, height of the villi and mucosa, width of the mucosa, and thickness of the submucosa and muscularis did not differ among the HSB fed the purified diets (P > 0.05). Additionally, these morphological variables did not differ significantly from HSB fed the control diet, 60% FM, as shown in the screenshots taken from the Concentriq software in **Figure 2**. The goblet cell count per villus did not exhibit a titratable trend among the HSB fed the purified diets. However, while HSB fed 0% Glu did exhibit the lowest distribution of goblet cells per villus (P < 0.05), this difference was not statistically significant compared to HSB supplemented with 1% Glu. Additionally, Dunnett’s test revealed that HSB not supplemented with Glu had significantly lower goblet cells per villus, decreasing by approximately 32% compared to HSB fed 60% FM. Goblet cell distribution is shown in **Figure 3**.

**Table 5:** Histological evaluation of gut morphology in the middle intestine of 100 g HSB after a 28-day supplemental period.

Variables	60% FM	0% Glu	1% Glu	3% Glu	5% Glu	8% Glu	^1^ *P-Values*
Radius of Gut (um)	927 ± 52.8	997 ± 43.2	1050 ± 83.0	1050 ± 35.3	1087 ± 56.5	947 ± 88.7	*0.593*
Height of Villus (um)	302 ± 15.2	382 ± 11.2	348 ± 25.7	361 ± 17.2	316 ± 30.8	352 ± 26.3	*0.388*
Height of Mucosa (um)	378 ± 16.4	451 ± 15.6	444 ± 31.5	462 ± 17.2	424 ± 32.7	438 ± 24.6	*0.862*
Width of Mucosa (um)	74.5 ± 2.54	77.2 ± 2.92	80.2 ± 3.29	84.0 ± 5.33	86.8 ± 3.05	75.9 ± 1.90	*0.176*
Submucosal Thickness (um)	51.6 ± 2.37	44.4 ± 4.23	48.2 ± 2.68	56.2 ± 3.61	51.3 ± 1.46	45.6 ± 3.60	*0.108*
Muscularis Thickness (um)	77.7 ± 6.40	62.1 ± 6.84^a^	58.9 ± 2.60^a^	79.5 ± 4.40^a^	66.8 ± 4.99^a^	59.5 ± 5.62^a^	*0.048*
Goblet Cell (Counts per Villus)	63.2 ± 4.49	43.0 ± 5.34^b^*	52.3 ± 4.93^a^^b^	59.1 ± 2.96^a^	57.2 ± 2.77^a^	60.2 ± 3.51^a^	*0.036*

Values are means ± SEM, n = 6 per diet.

^1^ Probability for one-way ANOVA for each AA among the purified diets in italics.

^a-c^ Means in a row without a common superscript letter differ (*P* < 0.05) as analyzed by one-way ANOVA and the SNK test.

^*^ Dunnet’s test used to compare effects of purified diets to 60% FM (*P* < 0.05).

## 4 Discussion

The aquaculture industry is moving away from FM based diets due to limited availability and skyrocketing prices. Over the past few decades, SBM has been a popular protein source for commercial feeding within the industry (39). It is paramount that teleost fish, like HSB, receive adequate AA provisions as it has been estimated that up to 85% of their energy requirements are provided by AAs (39, 59). Finding alternatives to protein composition in fish diets will benefit the industry, particularly for carnivorous fish, where they may not adequately obtain enough Glu from plant-based proteins compared to FM. Juvenile HSB are a useful model to assess the nutritional requirements of AAs such as Glu (47) and Gly (51, 60, 61) due to sensitive responses and relatively low costs. This study examined the effects of dietary Glu supplementation at various concentrations on growth, AA content within serum and intestinal mucosa, and intestinal morphology. While our previous study showed that a purified diet with +5% Glu supplementation, a total of 8% Glu, improved growth and goblet cell distribution, our study shows that this is not the case in 100 g HSB. findings. While these findings offer modest insights into glutamate supplementation in this stage of fish, they should be interpreted within the context of certain limitations, including sample size, time, and absence of direct immune markers.

Our experimental design used two fish per tank and with the lack of tools to tag them, we used the tank as the measurable unit for growth performance. HSB fed a purified diet showed no differences in growth parameters, nor did it improve compared to those fed FM. While the industry has established two to three phases for production, juvenile HSB may undergo multiple growth stages with varying AA requirements during these periods; for example, this study showed that 100 g HSB may not require Glu for growth. A study using a practical diet of similar-sized HSB by Gallagher showed there were no differences in weight gain compared to HSB fed FM after an 8-week feeding trial with various amounts of SBM as a replacement for FM (62). This study also observed no differences in growth between HSB <200 g and those fed 50% SBM in a 14-week dietary intervention, compared to those fed 100% FM (62). It is possible that these HSB of this size are not experiencing rapid growth and do not use AAs, like Glu, for growth. This was seen in 86.4 g rainbow trout (*Oncorhynchus mykiss*) that were supplemented with 2% Glu in a mixed protein diet with black soldier fly larvae meal (63). While using a purified diets did not improve growth compared to HSB fed FM, it was comparable. A study in Turbot (*Scophthalmus maximus*) with an initial BW of 31.8 g showed that these fish could have crystalline-AA replace 19% of dietary protein and not have adverse growth effects (64). Another study in rainbow trout with an initial BW of 48 g showed that the growth performance and feed efficiency improved with the single use of crystalline L-glutamic acid, glycine or glutamine; however, the mixture of all three showed the best results after 84 days of dietary intervention (65). More research is needed to compare growth performance between HSB fed purified diets and, more importantly, the inclusion of Glu in practical diets, such as those fed SBM, to assess the possibility of economic sustainability and applicability. For downstream experiments, only one fish from each tank was randomly selected for tissue collection, limiting the sample size for interpretation power. Feeding and social behavior could influence the fish represented in that tank and warrant a more cautious interpretation of the following results.

While our previous study was the first to report plasma AA concentrations in HSB fed a purified AA diet (47), this study will be the first to report serum AA concentrations in HSB fed a purified AA diet (**Table 3**) one hour postprandial. We opted to use serum to avoid the anticoagulant heparin, which would dilute our samples. In humans (66, 67) and dogs (68) serum and plasma AA concentrations are different, where serum samples are more concentrated, but exhibited similar trends. We observed that AA concentrations can differ between serum and plasma. For example, Hissen et al. (47) reported that in plasma, ornithine concentrations in 30 g HSB fed a total of 3% Glu had 40.6 ± 12.5 nmol/mL, while serum of 100 g HSB had undetectable levels. We also previously reported that HSB fed a total of 8% Glu had plasma ornithine concentrations of 39.1 ± 10.9 nmol/mL, in contrast to serum with 6.93 ± 2.85 nmol/mL. This could be age-dependent on usage for growth, but more studies are needed to verify this. For both studies, arginine and tyrosine were modulated, despite differences in concentration. Both studies showed that when HSB are supplemented with a total of 8% Glu arginine, concentrations increase (47). Our present study also showed that 5% Glu can produce the same effect, indicating the possibility that Glu may be converted into arginine. However, this has only been observed in the enterocytes of the small intestine of omnivorous mammals (69) and remains unknown in HSB. While not statistically significant in Hissen et al. (47), taurine plasma levels exhibited similar trends to this study’s serum levels where taurine levels decreased in HSB fed 8% Glu. As previously reported, taurine levels are approximately 1 mM (51, 60), which could indicate its use in producing glutathione (71).

To the authors’ knowledge, this is the first study to report intestinal mucosa AA concentrations for HSB fed a purified AA diet. Intriguingly, for this size HSB intestinal mucosa Glu concentrations increased with increasing dietary Glu levels, which, based on our previous study, could possibly be for mucin production through the abundance of goblet cells (47). However, our histological analysis from this current study showed that Glu supplementation did not improve intestinal morphology. Another possibility is that the increased availability of glycine and Glu within the intestinal mucosa of HSB fed 5% and 8% Glu could be used for glutathione synthesis (61, 70). Glu is metabolized into the tricarboxylic acid cycle (TCA cycle) by being converted into α-ketoglutarate (43, 72), which can supply ATP to enterocytes, supporting intestinal morphology (43, 73). It also modulates inflammatory and antioxidant responses supporting structural integrity through epithelial renewal and mucosal homeostasis (43, 74), but there appears to be a narrow optimal margin. This margin may be altered under inflammatory stress, such as with LPS seen in pigs, where dietary Glu improved intestinal physiology by enhancing mucosal structure through modulating Th17/Treg immune balance (74) and regulating gut-brain access through the corticotropin-releasing factor pathway (75). Further research is warranted to test immune parameters to elucidate acceptable margins for beneficial effects. We also observed that HSB fed 3% Glu had very low ornithine levels in the intestinal mucosa, which correlates with non-detectable levels in the serum. Ornithine is an immediate precursor for polyamine synthesis, which plays a role in intestinal mucosa regeneration in rats (76). Further study would be warranted to measure the enzyme ornithine decarboxylase activity in HSB to determine if ornithine is actively utilized in the intestinal mucosa, resulting in lower concentrations in both serum and intestinal mucosa. While we did not see differences in intestinal morphology among the HSB fed the purified diets and FM, we did observe HSB fed no Glu had a lower distribution of goblet cells per villus. This is a modest result but could indicate decreased mucosal immunity, as the cells are typical morphological markers of the mucosal immune response (77). In human and porcine intestinal cell lines, Glu has been shown to increase gut integrity (78, 79) and enhance the mucosal barrier function (80), thus without Glu, the intestinal health could be compromised due to the decrease in mucin production. We estimated the HSB villi to be approximately 0.04 mm^2^ and goblet cell counts averaging 55 cells per villus among all the diets, displaying a goblet cell density of about 1,375 per mm^2^, higher than that of other teleost fish including Tambaqui (*Colossoma macropomum*), which are omnivorous, and hybrid catfish (*Pseudoplatystoma reticulatum* × *Leiarius marmoratus*), which are carnivorous. A study done by Pereira et al., 2020 shows that with Alcian blue staining, the middle intestine of Tambaqui had goblet cells ranging from 35.59 to 58.07 per mm^2^ on the mucosa epithelium, while the hindgut exhibited approximately 18.43 per mm^2^ (81). The same study showed that the middle intestine of hybrid catfish had goblet cells ranging from 33.53 to 68.46 per mm^2^, while the hindgut had more at approximately 132.64 per mm^2^ (81). It is notable that the posterior intestine of the carnivorous fish had more goblet cells, and further studies are warranted to investigate dietary Glu’s impact on goblet cell distribution within this gut segment of HSB. This has also been observed in juvenile Asian sea bass exposed to intestinal coccidian by Thongrin et al. (82). They also discuss the concept of goblet cell hyperplasia, where, during a host response, goblet cells increase in the tissue to protect itself (82). However, Ladeira et al. (83) supplemented Glu using monosodium glutamate (MSG) in pacamã (*Lophiosilurus alexandri*), and observed an increase in goblet cell hyperplasia in fish fed diets containing 42.0 g/kg of MSG. This present study primarily focuses on modest morphology results, thus, it warrants future studies to determine whether Glu supplementation modulates a beneficial immune response within the mucosa by quantifying inflammatory and mucin expression, in addition to assessing barrier integrity.

In comparison to terrestrial animals, HSB seem to have a higher distribution of goblet cells. In a healthy mouse, there were 11–14 goblet cells per villus, and those numbers decreased to <10 with lower intestinal health (84). In piglets with a BW of approximately 5.6 kg, the duodenum, jejunum and ileum had approximately 4.7, 3.8 and 7.3 goblet cells per 100-μm length of villus, respectively (85). We estimate that HSB has approximately 15.1 goblet cells per 100-μm length; however, to make comparable remarks, villus heights for the villi in which goblet cells were counted were estimated because the intestinal tissue was intact and folds could not be fully extended. In these cases, folded villi were included to allow manual goblet cell enumeration per villus. To maintain comparability with this published work, these estimated heights of approximately 370 μm were used to generate a rough, order-of-magnitude comparison across studies. Another study with larger piglets with a BW of 12.5 kg had goblet cell counts ranging from 1.6 to 4.6 per 100-μm length in the villi while in the crypts it was 1.6 to 6.4 per 100-μm length within the duodenum (86). In the same study, the middle jejunum had goblet cell counts ranging from 0.2 to 2.6 per 100-μm length in the villi, and 0.2 to 4.2 per 100-μm length in the crypts (86). This shows that HSB have a larger distribution of goblet cells compared with piglets and mice. The study by Święch et al. (86) distinguished goblet cells with acidic and neutral pH mucin in pigs, which suggests the type of immune response presented through supplementation. A similar investigation should look into the effects of Glu supplementation on the types of goblet cells in HSB based on pH. Certain limitations should be taken into account when interpreting these findings. As mentioned, these 100 g HSBs hatched the spring 2024 and the trial was conducted spring 2025, making them just over a year old at the time of this study. Due to their size, our aquatic system may not be an ideal setup for this trial, as the fish had limited space for movement compared to what they would experience on a production farm. For investigators wishing to repeat this study, utilizing raceways, rectangular flowing-water tanks, whether on land, over ponds, or integrated into pond levees. This could have contributed to our poor FCR (~8.33), which are not ideal for the industry, where estimates with FM are <3.0 (87). When repeating this study, collecting plasma after 4-hours as described in (47) is necessary, for Plakas et al. (88) found that plasma free AA reached maximum total levels at 4 hours after feeding, while this present study collected serum samples one hour post-feeding. Further research is warranted to determine if free AAs in both serum and intestinal mucosa reach their maximal potential 4 hours post-feeding. This may explain why in this present study, ornithine levels were undetectable, while our previous study had a higher concentration of ornithine in the plasma (47). Additionally, one hour postprandial may not give the proper time for adequate digestion for FM, thus limiting all the comparisons between purified fed HSB and FM fed HSB. Future studies incorporating time points >4 hours would provide a more comprehensive comparison between HSB fed purified diets with Glu supplementation and those fed FM. Studies comparing AA concentrations in plasma and serum in humans (66, 67) and dogs (68) recommend utilizing plasma samples for analysis. Lastly, our intestinal samples for histology were intact, maintaining tissue integrity; however, butterflying the intestine to obtain straight, perpendicular longitudinal sections for embedding may be more ideal for assessing intestinal morphology. This method would be beneficial for obtaining more precise measurements of villus height and mucosal thickness, and for quantifying goblet cells per mm² or per 100 µm in length.

## 5 Conclusions

While using a purified AA diet is not the solution to alternative protein source use in aquatic diets, it provides precise control, allowing us to determine AA specific effects. In this study, we quantified growth parameters, AA concentrations in the serum and mucosa, and morphological parameters including goblet cell counts. Glu supplementation may not be beneficial for supporting growth in 100 g HSB, as they performed comparably to or worse than HSB fed 60% FM. Some AA concentrations were modulated in serum and mucosa with Glu supplementation; however, there is little evidence to suggest physiological utilization for the growth or intestinal health. However, a dietary Glu deficiency could reduce goblet cell distribution in the villi, potentially compromising mucosal immunity. These findings raise important considerations for the industry about whether Glu supplementation may be necessary across all growth stages of HSB, and research efforts may be more beneficial in investigating Glu supplementation in HSB weighing less than 50 g. It also illustrates the need for further investigation on immune parameters or the inclusion of Glu in practical diets such as SBM based diets.

## Data Availability

The original contributions presented in the study are included in the article/supplementary material. Further inquiries can be directed to the corresponding author.

## Glossary

AA: Amino acid
BW: Body weight
FAO: Food and Agriculture Organization of the United Nations's
FM: Fishmeal
Glu: Glutamate
NEAA: Non-essential amino acid
NRC: National Research Council
KHB: Krebs–Henseleit bicarbonate buffer
OPA: o-phthaldialdehyde
SBM: Soybean meal
SEM: Standard error of the mean
VMBS: Veterinary Medicine and Biomedical Sciences
WGR: Weight gain rate
